# Purification, biochemical characterization, and molecular cloning of cellulase from *Bacillus licheniform*is strain Z9 isolated from soil

**DOI:** 10.1186/s43141-022-00317-4

**Published:** 2022-02-22

**Authors:** Zainab E. Elsababty, Samir H. Abdel-Aziz, Atef M. Ibrahim, Adel A. Guirgis, Ghada E. Dawwam

**Affiliations:** 1 Botany and Microbiology Department, Faculty of Science, Benha University, Benha, 13518 Egypt; 2 Microbial Biotechnology Department, Genetic Engineering and Biotechnology Research Institute (GEBRI), University of Sadat City, P.O. Box 79, Menoufia, Egypt

**Keywords:** *Bacillus licheniformis*, Cellulases, Cloning, Biochemical characterization, Purification

## Abstract

**Background:**

Cellulose is the most prevalent biomass and renewable energy source in nature. The hydrolysis of cellulosic biomass to glucose units is essential for the economic exploitation of this natural resource. Cellulase enzyme, which is largely generated by bacteria and fungus, is commonly used to degrade cellulose. Cellulases are used in a variety of industries, including bioethanol manufacturing, textiles, detergents, drugs, food, and paper. As part of our quest to find an efficient biocatalyst for the hydrolysis of cellulosic biomass, we describe the amplification, cloning, and sequencing of cellulase (cel9z) from *Bacillus licheniformis* strain Z9, as well as the characterization of the resulting enzyme.

**Results:**

Cellulase was partially purified from *B. licheniformis* strain Z9 using (NH_4_)_2_SO_4_ precipitation and Sephadex G-100 gel column chromatography with 356.5 U/mg specific activity, 2.1-purification fold, and 3.07 % yield. The nucleotide sequence of the cellulase gene was deposited to the GenBank, *B. licheniformis* strain Z9 cellulase (cel9z) gene, under accession number MK814929. This corresponds to 1453 nucleotides gene and encodes for a protein composed of 484 amino acids. Comparison of deduced amino acids sequence to other related cellulases showed that the enzyme cel9z can be classified as a glycoside hydrolase family 9. SDS-PAGE analysis of the purified enzyme revealed that the molecular mass was 54.5 kDa. The optimal enzyme activity was observed at pH 7.4 and 30 °C. The enzyme was found to be strongly inhibited by Mg^2+^ and Na^+^, whereas strongly activated by Fe^3+^, Cu^2+^, and Ca^2+^.

**Conclusions:**

*B. licheniformis* strain Z9 and its cellulase gene can be further utilized for recombinant production of cellulases for industrial application.

**Supplementary Information:**

The online version contains supplementary material available at 10.1186/s43141-022-00317-4.

## Background

Cellulose is the most prevalent polysaccharide in nature and the primary component of plant cell walls [1]. A linear polymer of β-1,4-linked D-glucose residues makes up cellulose. Developing technologies for successful treatment and usage of cellulosic wastes as inexpensive carbon sources have been of substantial commercial importance. Cellulose is secreted by some bacterial species, like *Acetobacter, Rhizobium, Xanthococcus, Pseudomonas, Azotobacter, Aerobacter*, and *Alcaligenes,* in addition to being abundant in plants [2]. Due to the urgent need for green energy, cellulose has acquired economic interest in its hydrolysis bio-technique in recent decades. One of the most common methods for converting cellulose into reducing sugars, which can then be turned into ethanol and other compounds, is microbial hydrolysis [3].

Cellulases are a family of enzymes that catalyze the hydrolysis of cellulose to liberate glucose units [4]. Endoglucanase (EC 3.2.1.4), exoglucanase (EC 3.2.1.91), and β-glucosidase (EC 3.2.1.21) are the three primary components of the cellulase complex enzymes [5]. Endoglucanase works by cleaving intermolecular β-1,4-glycosidic bonds inside the cellulose chain to liberate oligosaccharides for exoglucanase and β-glucosidase to further hydrolyze [6]. Endoglucanase and exoglucanase create cello-oligosaccharides and cellobiose from cellulose, which are then converted to glucose by β-glucosidase [7]. Cellulases derived from fungi, bacteria, and yeasts have been studied extensively [3]. Different bacterial species as *Bacillus* [8], *Clostridium*, and *Ruminococcus* [9] have all been reported to produce cellulases. Cellulases are used in juice extraction processes, pulp and paper, textile industry, secondary metabolites, animal feed, extraction of vegetable dyes, and the production of fermentable sugars for biofuels [10]. Thus, the demand for this enzyme is increasing exponentially [11].

Cellulases were isolated and identified first only from culturable bacteria using a fermentation technique, and the whole cellulase potential of the site was not completely investigated. Due to the high substrate cost necessary for cellulase induction and the problems of maintaining the appropriate conditions for cellulase production, fermentation approaches have limitations [12]. As a result, recent breakthroughs in molecular approaches, such as the production of metagenomic libraries, will expand the pool of cellulolytic enzymes suitable for biofuel research, potentially solving these challenges. This new method will allow the extraction of cellulases and related enzymes from bacteria that are otherwise unculturable and may create novel enzymes with specialized applications [13]. Uncultured microorganisms make up a large part of the natural world’s biodiversity. Only 0.1–1% of the natural environment is made up of microorganisms that can be cultivated using conventional laboratory techniques [14]. Genes derived from metagenomic techniques have proven to be useful in identifying novel genes with specialized functions [15]. A new strategy for discovering novel enzymes is to clone and express the cellulase gene in an efficient host cell like *Escherichia coli* [16].

Our earlier studies based on isolation and identification of cellulolytic *B. licheniformis* strain Z9 from soils having the highest cellulolytic activity [17]. The present study is concerned with the amplification, sequencing, and cloning of the gene encoding cellulase. Also, biochemical characterization was investigated to determine the optimum enzyme activity. This research, combined with ongoing expression research, could lead to a low-cost system based on genetically recombinant *Escherichia coli* with industrial applications.

## Methods

### Bacterial strain

*B. licheniformis* strain Z9 (KT693282) was isolated from farm soil at Menoufia governorate, Egypt (30°35′50.09′′ North, 30°59′15.48′′ East) [17] and tested for its high performance for cellulase activity. The bacterial strain was cultured in nutrient broth and stored on nutrient agar at 4 °C and as 50% glycerol stocks at − 80 °C.

### Enzyme assay

The cellulase activity was observed by the 3, 5 dinitrosalicylic acid (DNS) method as described by Miller [18]. CMCase activity was determined by incubating 500 μl of 1% CMC in 50 mM sodium phosphate buffer (pH 7.2) with 500 μl cell free extract for 30 min at 50 °C. The reaction was stopped by adding 1 mL of 3, 5 dinitrosalicylic acid (DNS) reagent and incubated in a water bath for 10 min at 50 °C. After cooling at room temperature, the amount of glucose released was investigated with a spectrophotometer at 540 nm against a blank containing all the reagents minus the crude enzyme. A calibration curve for glucose was constructed to determine the CMCase activity. One unit (U) of cellulase activity was defined as the amount of enzyme that released 1 μmol of glucose per minute under the standard assay conditions. All assays were performed in triplicate.

### Protein assay

Protein concentration was estimated by the method of Bradford [19] using bovine serum albumin (BSA) as a standard against a blank was set with only distilled water.

### Molecular identification and DNA sequence analysis

To amplify the cellulase gene from the *B. licheniformis* strain Z9, degenerated gene-specific primers were designed complementary to the *B. licheniformis* strain SRCM100027 (CP021677) cellulase [ARW53264] gene sequence retrieved from the NCBI nucleotide database. This includes Forward primer Zf1:(5′ATGGCTTATTCTGCCGCAATCCTGTCA-3′) and reverse primer Zr1 (5′ GGCCATGTCGCTCTGCACGTAGTGG-3′). The PCR amplification reaction was performed in a total volume of 50 μl containing 2 μL of template DNA (50 ng/μL), 25 μL of 2X Taq PCR Master Mix (contains Taq DNA polymerase (0.05 U/μL), reaction buffer, 4 mM MgCl2, and 0.4 mM of each dNTP) provided by Thermo Fisher Scientific, USA, 2 μL of forward primer, 2 μL of reverse primer and 19 μL of Nuclease-free water. The following PCR conditions were used for amplification of cellulase gene: initial denaturation at 94 °C, 5 min, and 35 cycles of the following steps: denaturation at 94 °C, 30 s; annealing at 55 °C, 30 s; extension at 72 °C, 1 min; and final extension at 72 °C, 5 min. The amplified PCR products were checked on 1% agarose gel stained with ethidium bromide and visualized on a UV transilluminator. The Purified PCR products were cloned into pSC-A-amp/kan PCR Cloning Vector as recommended by the manufacturer (Stratagene, Agilent Technologies, USA). StrataClone SoloPack competent cells were used for the transformation and recovery of high-quality recombinant DNA. The purified PCR products were Sanger-sequenced with the BigDye terminator v3.1 sequencing kit and ABI PRISM® 3730xl Analyzer capillary sequencer (Applied Biosystems, Foster City, CA). Nucleotide sequences were determined on both strands of PCR amplification products at Macrogen Company, Seoul, South Korea. The nucleotide sequence data was assembled, analyzed with GENETYX computer software (Software Development Co. Ltd., Tokyo, Japan). The consensus sequence obtained was compared with other sequences available in the GenBank/NCBI database using the BLAST tool [20] and aligned using CLUSTAL O [21]. The sequence was deposited with the GenBank Data Library under accession number MK814929. The deduced amino acids sequence was analyzed with UniprotKB database Release 2020, Washington, USA (http:// UniprotKB.org/). The phylogenetic tree was drawn with MEGA11 software [22], using Kimura’s two-parameter model of sequence evolution. The robustness of the phylogenetic tree was estimated via bootstrap analysis using 1000 resampling.

### Conserved domain analysis and hydropathy plots of predicted cellulase cel9z

The protein sequences of cellulase cel9z were subjected to conserved domain analysis using the Conserved Domain Database tool of NCBI [23] (http://www.ncbi.nlm.nih.gov/Structure/cdd/). A hydropathy plot was generated using Expasy-Protscale (https://web.expasy.org/protscale/).

### Preparation of cell extracts

Cellulase enzyme was produced under submerged fermentation from isolated *B. licheniformis* strain Z9. Conical flasks containing 50 mL of a carboxymethylcellulose medium [24] were supplemented with 0.5% (w/v) of CMC at pH 7.2. The flasks were autoclaved at 121 °C for 20 min. These sterilized flasks were inoculated with 10% of an inoculum culture of *B. licheniformis* strain Z9 and incubated under agitation at 150 pm, for 72 h at 30 °C according to the method of MARCO [25]. Then, the culture was centrifuged at 10,000×*g* for 15 min at 4 °C to separate the cells. The clear cell-free supernatant (crude extract) was collected and concentrated by ultrafiltration using a MILTEX-HV ultrafiltration cell (Millipore, Ireland). The crude extract was stored at 4 °C and used for further analyses [26].

### Purification of cellulase

All steps were performed at 4 °C unless otherwise noted. The crude extract was saturated with (20–80%) ammonium sulphate with continuous stirring at 4 °C followed by centrifugation at 10,000×*g* for 15 min. Ammonium sulfate fraction (the developed pellet) was dialyzed against 50 mM sodium phosphate buffer (pH 7.2) for 6 h at 4 °C in a dialysis bag (20,000 kDa) and immersed in the same buffer at 4 °C overnight. Changing buffer at every 1 h intervals is important to achieve proper purification [27]. Fractions with high activity of cellulase were pooled together, dialyzed towards the above buffer, and concentrated by lyophilization (− 50 °C) for the next purification step. The dialysate was loaded onto a Sephadex G-100 column (2.5 × 40 cm) equilibrated with 50 mM sodium phosphate buffer (pH 7.2), and eluted in a gradient of NaCl (0–1 mol L^−1^) [25]. The cellulase was eluted from the column at a flow rate of 5 ml/min using the same buffer. Thirty-five fractions (5 ml each) were collected, dialyzed against the same buffer and the protein content was measured with a spectrophotometer at 280 nm. Fractions were checked for their purity by applying sodium dodecyl sulphate polyacrylamide gel electrophoresis (SDS-PAGE) technique.

### Sodium dodecyl sulfate-polyacrylamide gel electrophoresis analysis (SDS-PAGE)

To estimate the molecular weight of the partially purified enzyme, SDS-PAGE was done as reported by Laemmli [28]. Briefly, the SDS-PAGE gel slabs were prepared with upper 4% stacking gel and lower 10% resolving gel using a Bio-Rad electrophoresis system (Bio-Rad, CA, USA). The protein samples were mixed with sample buffer containing 62.5 mM Tris-HCl, pH 6.8, 25% glycerol, 0.01% bromophenol blue, 2% SDS, 10% b-mercaptoethanol, and then heated for 3 min before loading to the gel. The electrophoresis was carried out in running buffer (0.25 M Tris, 0.192 M glycine, 0.1% SDS, pH 8.3) and the gel was then stained by a solution of 0.15% Coomassie Brilliant Blue (CBB) R-250 in 50% ethanol and 10% glacial acetic acid. The samples were dissolved with sample buffer (50 mM Tris–HCl pH 6.8, 2% SDS, 10% glycerol, 1% β-mercaptoethanol, 0.01% bromophenol blue) and then applied to the wells, resolved by applying a constant current (100 V) across the gel. After the run, the resolved bands were visualized by Coomassie brilliant blue R-250 staining method. The molecular weights were estimated by comparing with standard broad range protein marker (iNtRON Biotechnology, Gangnam-STAIN™ Prestained Protein Ladder ranging from 10 to 245 kDa).

### Zymographic analysis

According to the method of Schwarz [29], zymograms were conducted to detect the proteins of cellulolytic activity from *B. licheniformis* strain Z9, with minor modifications as described below. A 0.2% CMC was added before polymerization to the resolving portion of 12% resolving gels except for no SDS and reducing agent were presented (native PAGE). For preventing aggregation, CMC was added slowly to the gel mixture while stirring. Gel polymerization was induced after all CMC was dissolved. Gels were allowed to polymerize overnight at room temperature, then kept at 4 °C until used (< 2 weeks). Solubilized protein samples were mixed with native sample buffer and then heated at 70 °C for 20 min to partially denature enzymes and reduce smearing of activity due to continuous enzymatic activity during electrophoresis. Following heating, the sample was briefly centrifuged to collect evaporated solution and loaded on gels for detection of cellulase activity. Electrophoresis was carried out at 4 °C at constant voltage (100 V) for approximately 4 h. For cellulolytic activity staining, gels were washed five times (6 min each) in 50 mL of washing buffer (50 mM phosphate buffer pH 7.2). The gel was incubated for 30 min at 37 °C in washing buffer without DTT to develop cellulase activity. Gels were then stained with 0.1% Congo Red for 15 min at room temperature and washed with 1 M NaCl until the clear cellulase band was visible. Gels were then immersed in 5% (v/v) acetic acid and photographed. The position of the cellulase enzyme on the gels was detected with a standard broad range protein marker (iNtRON Biotechnology, Korea).

### Characterization of the purified cellulase

#### Optimum temperature

To assess the effect of temperature on the enzyme activity, a reaction mixture of substrate and the partially purified enzyme was incubated at various temperatures. 100 μL of the appropriate concentration of enzyme was added to 100 μL of 1% CMC and completed to 1 mL with 800 μL of 20 mM glycine-NaOH buffer (pH 7.4), and incubated at 10 °C, 20 °C, 30 °C, 37 °C, 45 °C, and 60 °C temperature for 30 min. The non-incubated enzyme was used as the control (100%). The activity was then measured according to the method of Miller [18].

#### Optimum pH

This experiment was performed to investigate the effect of different buffers at different pH values on the partially purified cel9z protein. One hundred microliters of the appropriate concentration of enzyme was added to 100 μL of 1% CMC and completed to 1 mL with 800 μL of various buffers. The buffers were citrate phosphate (pH 4.2–7), Tris (Hydroxymethyl) aminomethane (Tris) (pH 7.2–9.0), and glycine-NaOH (9.5-10.6), and the activity was measured as described by Miller [18]. The non-treated enzyme activity was regarded as control (100%).

### Effect of metal ions and chemical reagents

For activators and/or inhibitors sensitivity studies, the partially purified enzyme was pre-incubated with a final concentration of 1mM of various metal ions and chemicals (Ag^+^, Na^+^, Cu^2+^, Co^2+^, Ca^2+^, Fe^3+^, Mg^2+^, SDS, and EDTA) dissolved in 50 mM Tris (Hydroxymethyl) aminomethane (pH 7.2) at 37 °C for 30 min. The activity assay in the absence of any chemical reagent or metal ions was recorded as a control (100%). The residual activity was measured by using the standard assay [18].

### Statistical analysis

Results were expressed as mean ± standard deviation and the data was analyzed using one-way ANOVA using GraphPad Prism for windows, www.graphpad.com.

## Results

### Molecular identification of cellulase gene

A total volume of 50 μL of PCR reaction was used for amplification of cellulase cel9z from *B. licheniformis* strain Z9. The length of the fragments was about 1500 bp. The PCR products of strain Z9 were sequenced to obtain an open reading frame (ORF) of 1453-bp gene sequence, which was consistent with the result of electrophoresis. This band was then excised, eluted, purified, and then subjected to cloning.

**Fig. 1:**
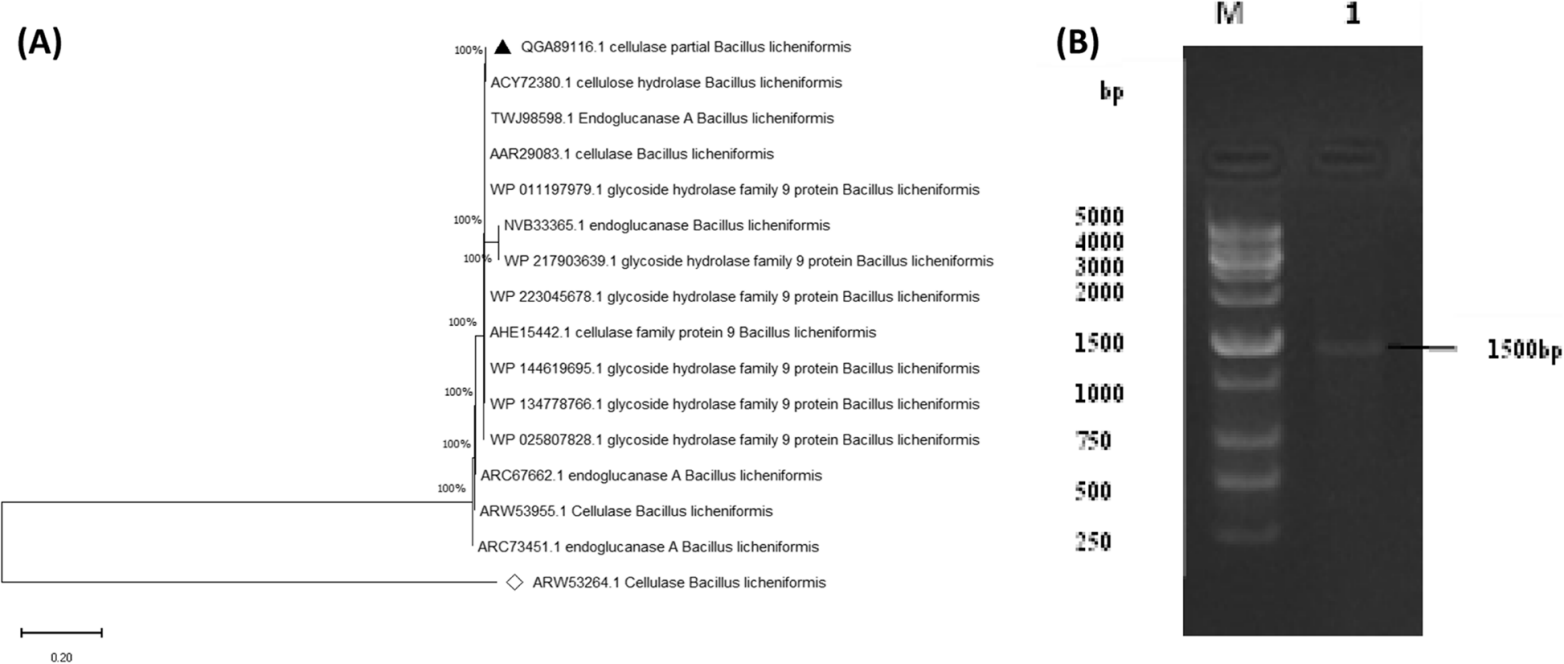
**A** Phylogenetic relationships of *Bacillus licheniformis* cel9z gene. The phylogenetic tree was drawn based on the neighbor joining method with Kimura 2 parameter distances using the MEGA 11 software. The values at the nodes represent the percentages of 1000 bootstrap replicates. Bar, 0.2 substitutions per nucleotide position. **B** Amplification of *B. licheniformis* Z9 cellulase gene. Lane M: SiZer-10 kb DNA ladder (iNtRON Biotechnology); lane 1: PCR product of *B. licheniformis* Z9 cellulase gene (1.5 kb)

The phylogenetic analysis of the cel9z and its related hits was carried out. Analysis revealed that the isolated *B. licheniformis* strain Z9 cellulase (cel9z) gene formed a distinct clade, containing endoglucanase and cellulase of *B. licheniformis* (Fig. 1A).

### Nucleotide sequence analysis

The obtained sequence was analyzed and compared with a sequence in the nucleotide database (NCBI) using the BLAST algorithm. The 1453 bp fragment of cel9z was fully sequenced at Macrogen Company, Seoul, South Korea, encoding a protein of 484 amino acids, with a calculated molecular mass of about 54.4 kDa as shown in Supplementary data 1.

### Analysis and alignment of cel9z protein sequences

The deduced amino acid sequence encoding cel9z was conducted from the sequence of nucleotides data. The amino acid sequence was aligned with the amino acid sequences of other organisms using UniProtKB program. The results obtained were shown in Supplementary data 2. The amino acid sequence comparison against protein databases indicated that (cel9z) shared over 97% similarity with their homologs. The highest sequence identity of cellulase (cel9z) was 99.7% % that compared with the Endoglucanase A of [*B. licheniformis*] (accession number TWK88936) which was defined by the whole genomic sequence.

Conserved domain analysis of cellulase cel9z protein revealed the presence of glycosyl hydrolase 9 domain of glycoside hydrolase 9 superfamily in the protein. The domain analysis found a specific hit for the with *e* value at 2.12e−165. Consequently, there was high confidence in the association between the protein query sequence and a conserved domain, resulting in a high confidence level for the inferred function of the protein query sequence. Further, hydropathy plots indicated that the protein was hydrophilic with a hydropathy score of − 3.

### Enzyme purification

**Fig. 2:**
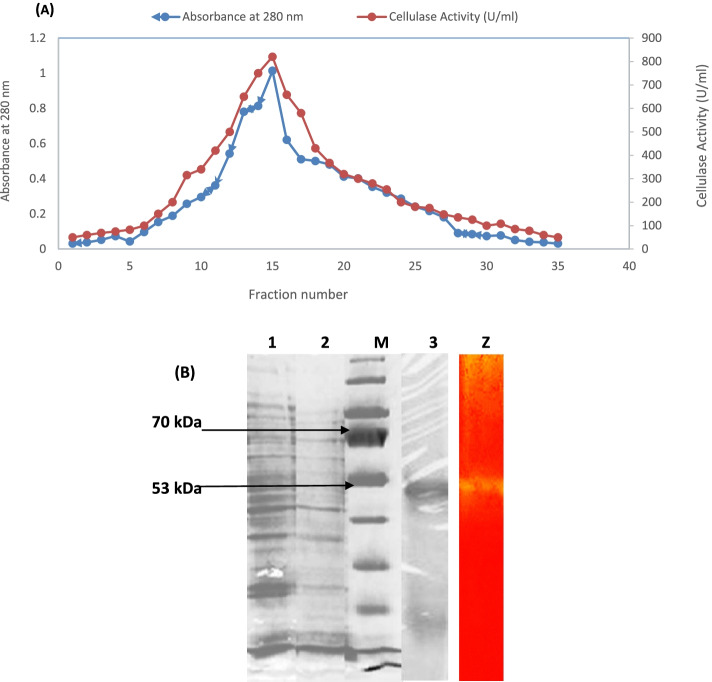
**A** purification profile of cellulase cel9z from *B. licheniformis* Z9 by Sephadex. G-100 column chromatography showing protein and enzyme activity. **B** SDS-PAGE and Zymogram analysis of cellulase enzyme from *B. licheniformis* Z9. lane (M): GangNam-STAIN™ Prestained Protein Ladder (iNtRON Biotechnology) of 10–245 kDa range; lane (1) crude protein; lane (2): proteins precipitated at 40–80% saturation of ammonium sulfate; lane (3): purified enzyme from Sephadex G-100 column; (Z) Zymogram

The purification profile of cellulase (cel9z) of *B. licheniformis* were listed in Table 1. Ammonium sulfate precipitation gave a purification fold of about 1.96 with a specific activity of 333.4 U/mg and 51.1% yield, while purification fold of about 2.1 and specific activity of 356.5 U/mg with 3.07% yield was achieved for gel filtration chromatography (Fig. 2A).

**Table 1:** Overall purification profile of cellulase from *Bacillus licheniformis* strain-Z9

Purification step	Volume (ml)^**a**^	Total protein (mg)^**b**^	Total activity (units)	Specific activity (U mg ^**–1**^ Protein min^**−1**^)	Purification (fold)^**c**^	Yield (%)^**d**^
Crude extract	150	157.5	26700	169.5	1	100
(NH_4_)_2_SO_4_ Precipitate	39	40.95	13650	333.4	1.96	51.1
Dialysis	20	9.5	3300	347.3	2.04	12.35
Gel exclusion chromatography (Sephadex G-100)	15	2.3	820	356.5	2.1	3.07

^a^,^b^ values represent the mean of the values from three independent experiments, with a standard deviation of < ± 5%

Specific activity = total activity/total protein

^c^Purification fold = specific activity of particular purification step/specific activity of the crude enzyme

^d^Yield (%) = (total activity of the particular purification step/total activity of the crude enzyme) × 100

### SDS-PAGE and zymogram analysis

Enzyme activity is concentrated and subjected to SDS-PAGE The enzyme cel9z showed a single band on SDS-PAGE with a molecular weight of approximately 54.4 kDa for both crude extract and partially purified enzyme. The enzymatic activity of partially purified cel9z is confirmed by CMC zymographic analysis. It is shown as a yellow halo against a red background (Fig. 2B).

Enzyme activity was concentrated and subjected to SDS-PAGE*.* The enzyme cel9z showed a single band on SDS-PAGE with a molecular weight of approximately 54.4 kDa for the partially purified enzyme. The enzymatic activity of purified cel9z was confirmed by CMC zymographic analysis. It was shown as a yellow halo against a red background (Fig. 2B).

### Biochemical characterization of cel9z

#### Screening of optimal temperature

**Fig. 3:**
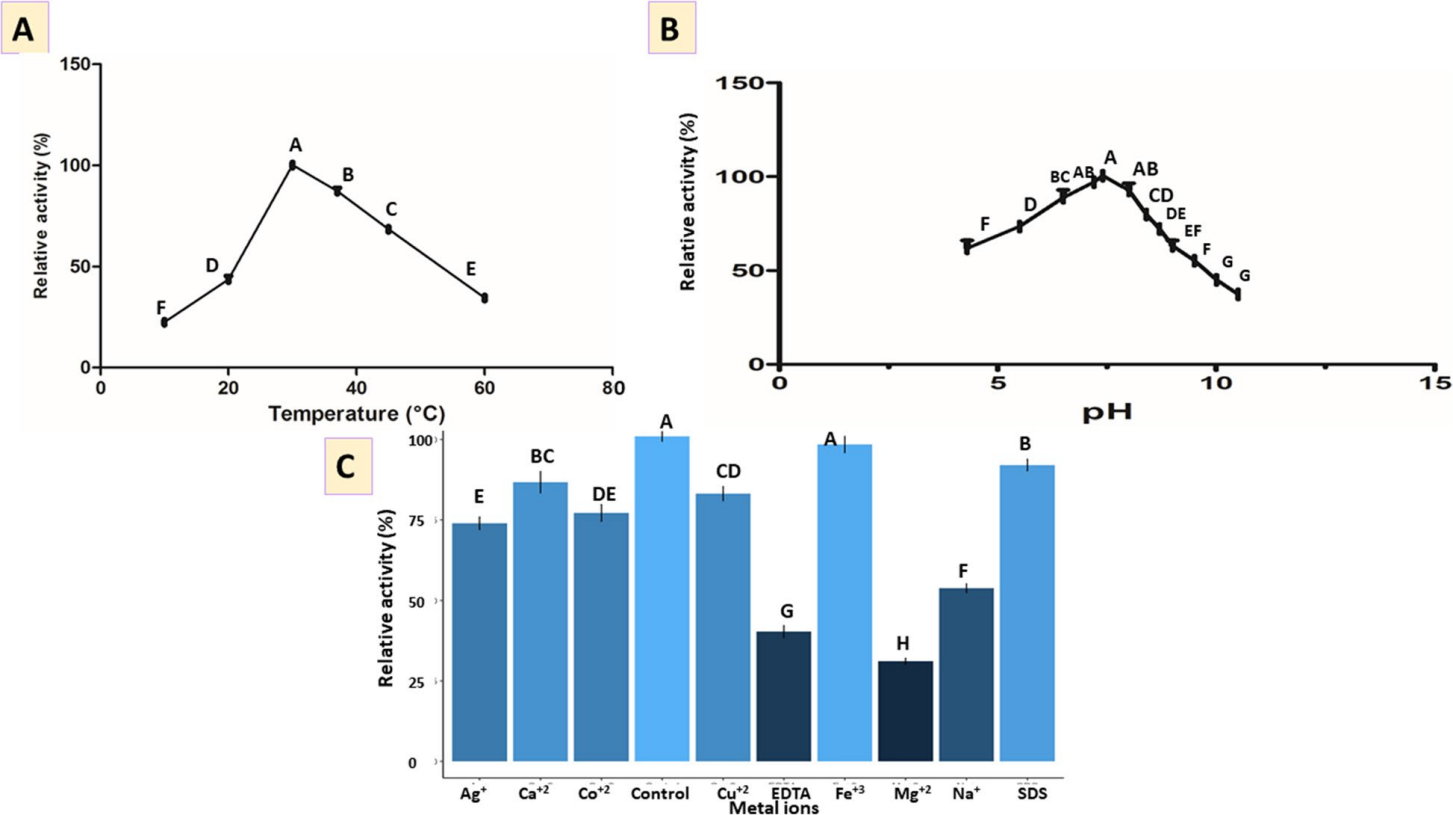
Effect of different physicochemical parameters on cel9z cellulase activity. **A** Temperature, **B** pH, **C** Metal ions and some chemical reagents. *Columns with different letters are significantly different from each other using Tukey’s post hoc test *P* value ˂ 0.05

The effect of temperature on the enzyme activity of cel9z from *B. licheniformis* was determined at various temperatures ranging from 10 to 60 °C as shown in (Fig. 3A). Results demonstrated that the optimum temperature of the enzyme was around 30 °C.

#### Screening of optimal pH

The effect of pH on the enzyme activity of cel9z from *B. licheniformis* was also examined at various pH levels ranging from pH 4.3 to pH 10.5. Cel9z exhibited the highest activity at pH 5.5–8, with an optimum pH of the enzyme activity at pH 7.4 (Fig. 3B).

#### Screening of the effect of metal ions and chemical reagents

The effect of metal ions and some chemical reagents at a final concentration of 1 mM (Ag^+^, Na^+^, Cu^2+^, Co^2+^, Ca^2+^, Fe^3+^, Mg^2+^, SDS, and EDTA) on cel9z enzyme activity from *B. licheniformis* strain Z9 was studied. The enzyme activity was strongly inhibited by Mg^2+^ and Na^+^ between 31 and 53.8% whereas Fe^3+^, Ca^2+^, and Cu^2+^ significantly activated the enzyme activity between 98.4 and 83.2% (Fig. 3C). Enzyme activity demonstrated a decrease by the chemical reagent EDTA by 40.3%, while SDS exhibited activation for the enzyme by 92%.

## Discussion

The increased demand for finding new sources of biofuels and renewable energy as an alternative to fossil fuels is one of the most common interests of researches worldwide. The enzymatic hydrolysis of cellulosic biomass by cellulases has been increased in several studies for their employment in many industrial applications. Different *Bacilli* species presented relevant results related to cellulase production [30]. In this study, gene encoding cellulase (cel9z) was derived from *B. licheniformis* strain-Z9 and was successfully amplified, purified, and sequenced. Cloning of powerful cellulase genes might be very important for the successful production and consequently industrial application of the enzyme. Furthermore, researchers have concentrated on producing recombinant cellulase. As a host, *E. coli* does not require any special media and grows rapidly. The key benefit of recombinant cellulase is that it may be scaled up to commercial levels without the usage of expensive substrates [31]. Cel9z from *B. licheniformis* strain Z9 has been cloned into the pSC-A-amp/kan PCR Cloning Vector, and further research into cel9z in a recombinant expression system is underway.

To provide pure and homogeneous cellulase for industrial applications, a comprehensive purification process is required. Therefore, the purification of crude enzyme extracts from *B. licheniformis* strain-Z9 was achieved through the combination of ammonium sulphate precipitation, 40–80% saturation followed by gel filtration on Sephadex G-100 with a total yield of 3.07% and 2.1-fold purification. Enhancement of specific activity is observed in each of the purification steps. In this respect, Azadian et al. [24] reported overall purification fold of the enzyme about 8.85 with the specific activity of 412.32 U/mg of cellulase from Bacillus sp. The purified enzyme was emerged as only one protein band with a molecular mass of 54.4 kDa on sodium dodecyl sulfate-polyacrylamide gel electrophoresis, confirming its purity.

The molecular weight 54.4 kDa of the partially purified enzyme is close to that reported by Assareh et al. [32]. Our results are also similar to those obtained by Rawat and Tewari [8] where cellulases of *Bacillus* species reveal a variable molecular mass ranging from 24.4 to 185 kDa.

CMCase production over a broad pH range is considered a characteristic of cellulases secreted by *Bacillus* sp. [33]. Similar results were obtained by Aygan et al. [34] who observed endoglucanases activity for the genus *Bacillus* at pH values from 8.5 to 10.0. In contrast, other studies have reported that the optimum pH for purified cellulase from *B. circulans* was 4.5 [35] while cellulase was produced by *Bacillus* sp. C14 was 11 [36].

The pH of the growth medium influences many enzymatic reactions by affecting the transport of chemical products and enzymes across the cell membrane [37]. A wide pH range is required for the application of enzymes in numerous conditions [3]. In the present study, the optimum pH was 7.4 and it was active over the broad pH region of 5.5–8. Obtained results are in good agreement with Pokhrel et al. [36] who reported that the optimum pH for the enzyme of *Bacillus subtilis* ranged between 6.5 and 7.5. Also, the optimum pH for *B.licheniformis* NLRI-X33 was 7.5 [38]. Other similar findings by Ekwealor et al. [39] showed very good activity at pH range 6 to 9.

The optimum temperature of the partially purified cellulase was found to be around 30 °C. The results are in close agreement with the study of *B. licheniformis* isolated from compost by Nallusamy et al. [40] at 37 °C, cellulase activity retained 87% activity. Also, the maximum endo (1,4) β-d-glucanase production from *B. licheniformis* KIBGE-IB2 was observed at 37 °C [41]. The same maximum cellulase activity at 37 °C of *B. pumilus* EWBCM1 isolated from the gut of earthworm was determined by Shankar and Isaiarasu [42]. There is a remarkable decrease of enzyme activity over 45 °C and retained only 42% activity at 60 °C. In this respect, Maurya et al. [43] reported that over a certain temperature, enzyme activity decreases with an increase in temperature because of enzyme denaturation. During the saccharification and fermentation processes, a low-temperature adaptation can save energy and money [3]. As a result, it can be used in a variety of industries, including food, feedstuffs, textiles, and pharmaceuticals [44]. According to current research, cel9z optimal activity occurs at a low temperature, which may be helpful for its prospective application.

The partially purified cellulase was also screened to determine its enzymatic activity in the presence of different metal ions Ag^+^, Na^+^, Cu^2+^, Co^2+^, Ca^2+^, Fe^3+^, Mg^2+^, SDS, and EDTA. The cellulase activity was found to be enhanced in the presence of Fe^3+^, Cu^2+^, and Ca^2+^. However, the enzyme was inhibited by the presence of metal ions Mg^2+^, and Na^+^. The opposite result was reported by Azadian et al. [24], where the CMCase activity was enhanced in the presence of Mg2+ (110%). The inhibition by the same divalent cations was also reported in cellulase enzymes from *B. licheniformis* strain MK7 and Bacillus amyoliquefaciens DL-3 [45]. The inhibitory effect of Mg^2+^ in this study is contrary to the work of Ekwealor et al. [39] who reported the stimulatory effect of MgSO_4_ on the activity of CMCase. Reports also revealed that metal ion in the form of a salt such as CaCl_2_·6H_2_O provides protection to some enzymes against thermal denaturation and plays an important role to stabilize the native forms at high temperatures [32]. The inhibition of cellulase by Mg^2+^ and Na^+^ ions may be linked to the competition between the exogenous cations and the protein-associated cations, resulting in a decreased metalloenzyme activity. EDTA was found to be inhibitory to the activity of cellulase used in this study. Low concentration of low valent metal ions had almost no inhibition effects on enzyme activity. Therefore, employing cel9z in the industry is promising.

Currently, some developed studies were applied to produce industrial cellulase by *Bacilli* strains due to the high bacterial growth, compared to fungi, and their ability to adapt to low-cost carbon sources, such as sugar cane bagasse. In addition, bacterial cellulase is considered a potent enzyme for the application in second-generation ethanol produced from sugarcane biomass in Brazil [46]. Further studies of the cellulase gene (cel9z) will provide insights into the function of the protein and its biochemical properties.

## Conclusion

In the current study, successful cloning of the gene-producing cellulase appears to be a viable technique that will lead to the creation of a low-cost effective strategy for achieving considerable lignocellulosic waste bioconversions. Furthermore, the purified cellulase (cel9z) from *B. licheniformis* Z9 showed a wide pH and temperature range. Further expression of the cloned gene will reveal information regarding the function of the produced protein, as well as its biochemical properties and prospective industrial applications.

## Supplementary Information

**Additional file 1: Supplementary data 1.** The nucleotide sequence of Bacillus licheniformis strain Z9 recombinant cellulase (cel9z) gene and its deduced amino acid residues. **Supplementary data 2.** Multiple sequence alignment of Bacillus licheniformis (Cel9z) with other glycosyl hydrolase family QGA89116: B. licheniformis (Cel9z) deduced amino acid sequence. ARW53264: Cellulase [B. licheniformis]; NVB33365: endoglucanase [B. licheniformis] ; WP_217903639: glycoside hydrolase family 9 protein [B. licheniformis]; ARC67662: endoglucanase A [ B. licheniformis]; ARW53264: Cellulase [B. licheniformis]; ARC73451: endoglucanase A [B. licheniformis]; WP_025807828: glycoside hydrolase family 9 protein [B. licheniformis]; WP_144619695: glycoside hydrolase family 9 protein [B. licheniformis]; TWJ98598: Endoglucanase A [B. licheniformis]; WP_134778766: glycoside hydrolase family 9 protein [B. licheniformis]; WP_011197979: endoglucanase [B. licheniformis]; WP_223045678: glycoside hydrolase family 9 protein [B. licheniformis]; AAR29083: cellulase [B. licheniformis]; AAR29083: cellulase [B. licheniformis] AHE15442: cellulase family protein 9 [B. licheniformis]; ACY72380: cellulose hydrolase [B. licheniformis].

## Data Availability

All data generated or analyzed during this study are included in this article.

## Abbreviations

BSA: Bovine serum albumin
Cel9z: Cellulase of Bacillus licheniformis strain Z9
CMC: Carboxymethylcellulose
DNA: Deoxynucleic acid
DNS: Dinitrosalicylic acid
EC: Enzyme classification
EDTA: Ethylene diamine tetra acetic acid
ORFs: Open reading frame
PCR: Polymerase chain reaction
SDS-PAGE: Sodium dodecyl sulphate-polyacrylamide gel electrophoresis
16S rRNA: 16S ribosomal ribonucleic acid

## References

[1] Singhania RR, Sukumaran RK, Patel AK, Larroche C, Pandey A (2010). Advancement and comparative profiles in the production technologies using solid-state and submerged fermentation for microbial cellulases. Enzyme Microb Technol.

[2] Gao M, Li J, Bao Z, Hu M, Nian R, Feng D et al (2019) A natural in situ fabrication method of functional bacterial cellulose using a microorganism. Nat Commun 10. 10.1038/s41467-018-07879-3 10.1038/s41467-018-07879-3 PMC6347598 30683871

[3] Ma L, Aizhan R, Wang X, Yi Y, Shan Y, Liu B (2020). Cloning and characterization of low-temperature adapted GH5-CBM3 endo-cellulase from Bacillus subtilis 1AJ3 and their application in the saccharification of switchgrass and coffee grounds. AMB Express.

[4] Nishida Y, Suzuki K, Kumagai Y, Tanaka H, Inoue A, Ojima T (2007). Isolation and primary structure of a cellulase from the Japanese sea urchin Strongylocentrotus nudus. Biochimie.

[5] Immanuel G, Dhanusha R, Prema P, Palavesam A (2006). Effect of different growth parameters on endoglucanase enzyme activity by bacteria isolated from coir retting effluents of estuarine environment. Int J Environ Sci Technol.

[6] Lynd L, Weimer PJ, van Zyl W, Pretorius I (2022) Microbiol Mol Biol Rev. 10.1128/MMBR.66.4.739.2002 10.1128/MMBR.66.3.506-577.2002 PMC120791 12209002

[7] Mohanram S, Amat D, Choudhary J, Arora A, Nain L (2013) Novel perspectives for evolving enzyme cocktails for lignocellulose hydrolysis in biorefineries. Sustain Chem Process 1. 10.1186/2043-7129-1-15

[8] Rawat R, Tewari L (2012). Purification and characterization of an acidothermophilic cellulase enzyme produced by Bacillus subtilis strain LFS3. Extremophiles.

[9] Edward B, Shoham Y, Lamed R (2006). Lignocellulose-decomposing bacteria and their enzyme systems. Prokaryotes.

[10] Singh R, Kumar M, Mittal A, Mehta P (2016) Microbial enzymes: industrial progress in 21st century. 3 Biotech 6. 10.1007/s13205-016-0485-8 10.1007/s13205-016-0485-8 PMC4991975 28330246

[11] Jayasekara S, Ratnayake R (2019). Microbial cellulases: an overview and applications.

[12] Vadala B, Deshpande S, Deshpan A (2021) Soluble expression of recombinant active cellulase in E.coli using B.subtilis (natto strain) cellulase gene. J Genet Eng Biotechnol 19. 10.1186/s43141-020-00103-0 10.1186/s43141-020-00103-0 PMC7801579 33428026

[13] Maki M, Leung K, Qin W (2009). The prospects of cellulase-producing bacteria for the bioconversion of lignocellulosic biomass. Int J Biol Sci.

[14] Ravin N, Mardanov A, Skryabin K (2015). Metagenomics as a tool for the investigation of uncultured microorganisms. Russ J Genet.

[15] Alves L, Westmann C, Lencioni Lovate G, de Siqueira G, Borelli T, Guazzaroni M-E (2018). Metagenomic approaches for understanding new concepts in microbial science. Int J Genomics.

[16] Petridis L, Smith J (2018) Molecular-level driving forces in lignocellulosic biomass deconstruction for bioenergy. Nat Rev Chem 2. 10.1038/s41570-018-0050-6

[17] Abdel-Aziz SH, Ibrahim AM, Guirgis AA, Dawwam GE, Elsababty ZE (2021). Isolation and screening of cellulase producing bacteria isolated from soil. Benha J Appl Sci.

[18] Miller GL (1959) Use of dinitrosalicylic acid reagent for detection of reducing sugars. Anal Chem 31. 10.1021/ac60147a030

[19] Bradford MM (1976). A rapid and sensitive method for quantitation of microgram quantities of protein utilizing the principle of protein-dye binding. Eur J Anaesthesiol.

[20] Altschul S, Madden TL, Schaffer A, Zhang J, Zhang Z, Miller WE (1997). Gapped BLAST and PSI-BLAST: a new generation of protein databases search programs. Nucleic Acids Res.

[21] Thompson J, Higgins DG, Gibson IJ (1994). CLUSTAL W: improving the sensitivity of progressive multiple sequence alignment through sequence weighting, position-specific gap penalties and weight matrix choice. Nucleic Acids Res.

[22] Kumar S, Stecher G, Tamura K (2016). MEGA7: molecular evolutionary genetics analysis version 7.0 for bigger datasets. Mol Biol Evol.

[23] Lu S, Wang J, Chitsaz F, Derbyshire M, Geer R, Gonzales N et al (2019) CDD/SPARCLE: the conserved domain database in 2020. Nucleic Acids Res 48. 10.1093/nar/gkz991 10.1093/nar/gkz991 PMC6943070 31777944

[24] Azadian F, Badoei Dalfard A, Namaki-Shoshtari A, Karami Z, Hassanshahian M (2017) Production and characterization of an acido-thermophilic, organic solvent stable cellulase from Bacillus sonorensis HSC7 by conversion of lignocellulosic wastes. J Genet Eng Biotechnol 15. 10.1016/j.jgeb.2016.12.005 10.1016/j.jgeb.2016.12.005 PMC6296611 30647655

[25] Marco É, Heck K, Martos E, van der Sand S (2017) Purification and characterization of a thermostable alkaline cellulase produced by Bacillus licheniformis 380 isolated from compost. An Acad Bras Cienc 89. 10.1590/0001-3765201720170408 10.1590/0001-3765201720170408 29044330

[26] Ramachandra M, Crawford D, Pometto A (1988). Extracellular enzyme activities during lignocellulose degradation by Streptomyces spp.: a comparative study of wild-type and genetically manipulated strains. Appl Environ Microbiol.

[27] Krishnan A, Kumar G, Loganathan K, Rao B (2011). Optimization, production and partial purification of extracellular α-amylase from Bacillus sp. Marini. Arch Appl Sci Res.

[28] Laemmli U (1970). Cleavage of structural proteins during assembly of head of bacteriophage-T4. Nature.

[29] Schwarz W, Bronnenmeier K, Gräbnitz F, Staudenbauer W (1987). Activity staining of cellulases in polyacrylamide gels containing mixed linkage β-glucans. Anal Biochem.

[30] Gaur R, Tiwari S (2015). Isolation, production, purification and characterization of an organic-solvent-thermostable alkalophilic cellulase from Bacillus vallismortis RG-07. BMC Biotechnol.

[31] Kim D, Ku S (2018) Bacillus cellulase molecular cloning, expression, and surface display on the outer membrane of Escherichia coli. Molecules 23. 10.3390/molecules23020503 10.3390/molecules23020503 PMC6017809 29495265

[32] Assareh R, Zahiri H, Noghabi K, Aminzadeh S, Bakhshi Khaniki G (2012). Characterization of the newly isolated Geobacillus sp T1, the efficient cellulase-producer on untreated barley and wheat straws. Bioresour Technol.

[33] Mawadza C, Hatti-Kaul R, Zvauya R, Mattiasson B (2000). Purification and characterization of cellulases produced by two Bacillus strains. J Biotechnol.

[34] Aygan A, Karcioglu Batur L, Arikan B (2011). Alkaline thermostable and halophilic endoglucanase from Bacillus licheniformis C108. Afr J Biotechnol.

[35] Kim C (1995). Characterization and substrate specificity of an Endo-b-1,4-D- Glucanase I (Avicelase I) from an extracellular multienzyme complex of Bacillus circulans. Appl Environ Microbiol.

[36] Pokhrel B, Bashyal B, Thapa Magar R (2014). Production, purification and characterization of cellulase from Bacillus subtilis isolated from soil. Eur J Biotechnol Biosci.

[37] Liang Y, Feng Z, Yesuf J, Blackburn J (2009). Optimization of growth medium and enzyme assay conditions for crude cellulases produced by a novel thermophilic and cellulolytic bacterium, Anoxybacillus sp. 527. Appl Biochem Biotechnol.

[38] Tae-Il K, Han JD, Jeon BS, Yang CB, Kim KN, Kim MK (2000). Isolation from cattle manure and characterisation of Bacilluslicheniformis NLRI-X33 secreting cellulase. Asian-Australas J Anim Sci.

[39] Ekwealor C, Odibo F, Onwosi C (2017). Partial purification and characterization of cellulase produced by Bacillus sphaericus CE-3. Adv Microbiol.

[40] Nallusamy S, Amira A, Al-Bahry S, Abdulkhadir E, Eltayeb E (2016). Isolation and characterization of cellulolytic Bacillus licheniformis from compost. Afr J Biotechnol.

[41] Karim A, Nawaz M, Aman A, Ul Qader SA (2014) Hyper production of cellulose degrading endo (1,4) β-d-glucanase from Bacillus licheniformis KIBGE-IB2. J Radiat Res Appl Sci. 10.1016/j.jrras.2014.06.004

[42] Shankar T, Isaiarasu L (2011). Cellulase production by Bacillus pumilus EWBCM1 under varying cultural conditions. Middle East J Sci Res.

[43] Maurya DP, Singh D, Pratap D, Maurya JP (2012). Optimization of solid state fermentation conditions for the production of cellulase by Trichoderma reesei. J Environ Biol.

[44] Vester J, Glaring M, Stougaard P (2014). Discovery of novel enzymes with industrial potential from a cold and alkaline environment by a combination of functional metagenomics and culturing. Microb Cell Factories.

[45] Lee Y-J, Kim B-K, Lee B-H, Jo K-I, Lee N-K, Chung C-H (2008). Purification and characterization of cellulase produced by Bacillus amyoliquefaciens DL-3 utilizing rice hull. Bioresour Technol.

[46] Ladeira S, Cruz E, Delatorre A, Batista Barbosa J, Martins M (2015) Cellulase production by thermophilic Bacillus sp. SMIA-2 and its detergent compatibility. Electron J Biotechnol 33. 10.1016/j.ejbt.2014.12.008

