# Supplementary material for: Purification, biochemical characterization, and molecular cloning of cellulase from Bacillus licheniformis strain Z9 isolated from soil
Source: J Genet Eng Biotechnol. 2022 Feb 22;20:34. doi: 10.1186/s43141-022-00317-4 (PMC8864052; doi:10.1186/s43141-022-00317-4)
Supplement: Supplementary file 1 — Additional file 1: Supplementary data 1. The nucleotide sequence of Bacillus licheniformis strain Z9 recombinant cellulase (cel9z) gene and its deduced amino acid residues. Supplementary data 2. Multiple sequence alignment of Bacillus licheniformis (Cel9z) with other glycosyl hydrolase family QGA89116: B. licheniformis (Cel9z) deduced amino acid sequence. ARW53264: Cellulase [B. licheniformis]; NVB33365: endoglucanase [B. licheniformis] ; WP_217903639: glycoside hydrolase family 9 protein [B. licheniformis]; ARC67662: endoglucanase A [ B. licheniformis]; ARW53264: Cellulase [B. licheniformis]; ARC73451: endoglucanase A [B. licheniformis]; WP_025807828: glycoside hydrolase family 9 protein [B. licheniformis]; WP_144619695: glycoside hydrolase family 9 protein [B. licheniformis]; TWJ98598: Endoglucanase A [B. licheniformis]; WP_134778766: glycoside hydrolase family 9 protein [B. licheniformis]; WP_011197979: endoglucanase [B. licheniformis]; WP_223045678: glycoside hydrolase family 9 protein [B. licheniformis]; AAR29083: cellulase [B. licheniformis]; AAR29083: cellulase [B. licheniformis] AHE15442: cellulase family protein 9 [B. licheniformis]; ACY72380: cellulose hydrolase [B. licheniformis]. [file 43141_2022_317_MOESM1_ESM.docx]

**Supplementary Data 1. The nucleotide sequence of *Bacillus licheniformis* strain -Z9 cellulase (cel9z) gene and its deduced amino acid residues.**

V W H F * * S S L * A * R R F Q K R P V Q L L L K N I

1 GTTTGGCACTTTTAGTGATCTTCTCTATGAGCATAGCGTCGTTTTCAGAAAAGACCCGTGCAGCTTCTGCTGAAGAATAT 80

1 CAAACCGTGAAAATCACTAGAAGAGATACTCGTATCGCAGCAAAAGTCTTTTCTGGGCACGTCGAAGACGACTTCTTATA 80

L I I M L N C C K S L C Y F M K H S A R E D F R K T

81 CCTCATAATTATGCTGAACTGCTGCAAAAGTCTTTGTTATTTTATGAAGCACAGCGCTCGGGAAGACTTCCGGAAAACAG 160

81 GGAGTATTAATACGACTTGACGACGTTTTCAGAAACAATAAAATACTTCGTGTCGCGAGCCCTTCTGAAGGCCTTTTGTC 160

A G * I G E E T P G L R T E K T L A S I * R E G G M M

161 CCGGCTGAATTGGAGAGGAGACTCCGGGCTTGAGGACGGAAAAGACGTTGGCCTCGATTTAACGGGAGGGTGGTATGATG 240

161 GGCCGACTTAACCTCTCCTCTGAGGCCCGAACTCCTGCCTTTTCTGCAACCGGAGCTAAATTGCCCTCCCACCATACTAC 240

P A T T * S S V C R W L I L P Q S C H G R S M S T Q M

241 CCGGCGACCACGTGAAGTTCGGTCTGCCGATGGCTTATTCTGCCGCAATCCTGTCATGGTCGGTCTATGAGTACCCAGAT 320

241 GGCCGCTGGTGCACTTCAAGCCAGACGGCTACCGAATAAGACGGCGTTAGGACAGTACCAGCCAGATACTCATGGGTCTA 320

P T K N R V S L M R R W T I L N G R Q T T F L K P I

321 GCCTACAAAGAATCGGGTCAGCTTGATGCGGCGCTGGACAATATTAAATGGGCGACAGACTACTTTCTTAAAGCCCATAC 400

321 CGGATGTTTCTTAGCCCAGTCGAACTACGCCGCGACCTGTTATAATTTACCCGCTGTCTGATGAAAGAATTTCGGGTATG 400

R L L M N C G A K S E M A L * T T H G G G R P K * C R

401 GGCTCCTTATGAATTGTGGGGCCAAGTCGGAAATGGCGCTCTAGACCACGCATGGTGGGGGCCGGCCGAAGTAATGCCGA 480

401 CCGAGGAATACTTAACACCCCGGTTCAGCCTTTACCGCGAGATCTGGTGCGTACCACCCCCGGCCGGCTTCATTACGGCT 480

* S A L P I R S M P A V R G Q T L L V V Q P Q R * H Q

481 TGAAGCGCCCTGCCTATAAGATCGATGCCGGCTGTCCGGGGTCAGACCTTGCTGGTGGTACAGCCGCAGCGCTAGCATCA 560

481 ACTTCGCGGGACGGATATTCTAGCTACGGCCGACAGGCCCCAGTCTGGAACGACCACCATGTCGGCGTCGCGATCGTAGT 560

H Q L F S S R Q I L L T L K N Y W L M P S N C M I L

561 GCATCAATTATTTTCAAGCCGACAGATTCTTCTTACTCTGAAAAATTACTGGCTCATGCCAAGCAATTGTATGATTTTGC 640

561 CGTAGTTAATAAAAGTTCGGCTGTCTAAGAAGAATGAGACTTTTTAATGACCGAGTACGGTTCGTTAACATACTAAAACG 640

P T A T A A N I Q T A L Q T H S N I I I R G A G I K M

641 CGACCGCTACCGCGGCAAATATTCAGACTGCATTACAGACGCACAGCAATATTATAATTCGTGGAGCGGGTATAAAGATG 720

641 GCTGGCGATGGCGCCGTTTATAAGTCTGACGTAATGTCTGCGTGTCGTTATAATATTAAGCACCTCGCCCATATTTCTAC 720

N * H G E L S G S T W Q Q K N N N I W I K P L L R S Q

721 AACTGACATGGGGAGCTGTCTGGCTCTACTTGGCAACAGAAGAACAACAATATTTGGATAAAGCCCTTGCTTCGGTCTCA 800

721 TTGACTGTACCCCTCGACAGACCGAGATGAACCGTTGTCTTCTTGTTGTTATAAACCTATTTCGGGAACGAAGCCAGAGT 800

I G A I P Q T G L T A G R F P G M T S L T E H S C C

801 GATTGGGGCGATCCCGCAAACTGGCCTTACCGCTGGACGCTTTCCTGGGATGACGTCACTTACGGAGCACAGCTGCTGCT 880

801 CTAACCCCGCTAGGGCGTTTGACCGGAATGGCGACCTGCGAAAGGACCCTACTGCAGTGAATGCCTCGTGTCGACGACGA 880

S L V * Q T I P V L S N L S N A I L I I G R Q A T V I

881 CGCTCGTCTGACAAACGATTCCCGTTTTGTCAAATCTGTCGAACGCAATCTTGATTATTGGTCGACAGGCTACAGTCATA 960

881 GCGAGCAGACTGTTTGCTAAGGGCAAAACAGTTTAGACAGCTTGCGTTAGAACTAATAACCAGCTGTCCGATGTCAGTAT 960

M E A * N G S R I R R A V W P G L S S G D H C D T L R

961 ATGGAAGCATAGAACGGATCACGTATACGCCGGGCGGTTTGGCCTGGCTTGAGCAGTGGGGATCATTGCGATACGCTTCG 1040

961 TACCTTCGTATCTTGCCTAGTGCATATGCGGCCCGCCAAACCGGACCGAACTCGTCACCCCTAGTAACGCTATGCGAAGC 1040

M P L F S L S F I P I G W I Q K K R K D I G I L L F

1041 AATGCCGCTTTTCTCGCTTTCGTTTATTCCGATTGGGTGGATACAGAAAAAGCGAAAAGATATCGGGATTTTGCTGTTCG 1120

1041 TTACGGCGAAAAGAGCGAAAGCAAATAAGGCTAACCCACCTATGTCTTTTTCGCTTTTCTATAGCCCTAAAACGACAAGC 1120

G K R S I C * E I I R S S E A L S L D T V K I R R N I

1121 GCAAACGGAGTATATGCTAGGAGATAATCCGCAGCAGCGAAGCTTTGTCGTTGGATACGGTAAAAATCCGCCGAAACATC 1200

1121 CGTTTGCCTCATATACGATCCTCTATTAGGCGTCGTCGCTTCGAAACAGCAACCTATGCCATTTTTAGGCGGCTTTGTAG 1200

R I T V Q H T V H G P I R * M C L K T I A I P Y T A H

1201 CGCATCACCGTACAGCACACGGTTCATGGGCCAATCAGATGAATGTGCCTGAAAACCATCGCCATACCCTATACGGCGCA 1280

1201 GCGTAGTGGCATGTCGTGTGCCAAGTACCCGGTTAGTCTACTTACACGGACTTTTGGTAGCGGTATGGGATATGCCGCGT 1280

* S A V R E G T I R T E M T * Q I M R Q T K L R S I

1281 TTAGTCGGCGGTCCGGGAAGGGACGATTCGTACCGAGATGACATAACAGATTATGCGTCAAACGAAGTTGCGATCGATTA 1360

1281 AATCAGCCGCCAGGCCCTTCCCTGCTAAGCATGGCTCTACTGTATTGTCTAATACGCAGTTTGCTTCAACGCTAGCTAAT 1360

I M P L L P A T * R K C F S C S G K A M F R C L I F R

1361 TAATGCCGCTTTTACCGGCAACGTAGCGAAAATGTTTCAGCTGTTCGGGAAAGGCCATGTTCCGCTGCCTGATTTTCCGG 1440

1361 ATTACGGCGAAAATGGCCGTTGCATCGCTTTTACAAAGTCGACAAGCCCTTTCCGGTACAAGGCGACGGACTAAAAGGCC 1440

R R K H

1441 AGAAGGAAACACC 1453

1441 TCTTCCTTTGTGG 1453

**Supplementary Data 2. Multiple sequence alignment of *Bacillus licheniformis* (Cel9z) with other glycosyl hydrolase family QGA89116: *B. licheniformis* (Cel9z) deduced amino acid sequence. ARW53264: Cellulase [*B. licheniformis*]; NVB33365: endoglucanase [*B. licheniformis*] ; WP_217903639: glycoside hydrolase family 9 protein [*B. licheniformis*]; ARC67662: endoglucanase A [*B. licheniformis*]; ARW53264: Cellulase [*B. licheniformis*]; ARC73451: endoglucanase A [*B. licheniformis*]; WP_025807828: glycoside hydrolase family 9 protein [*B. licheniformis*]; WP_144619695: glycoside hydrolase family 9 protein [*B. licheniformis*]; TWJ98598: Endoglucanase A [*B. licheniformis*]; WP_134778766: glycoside hydrolase family 9 protein [*B. licheniformis*]; WP_011197979: endoglucanase [*B. licheniformis*]; WP_223045678**: **glycoside hydrolase family 9 protein [*B. licheniformis*]; AAR29083: cellulase [B. licheniformis]; AAR29083: cellulase [*B. licheniformis*] AHE15442: cellulase family protein 9 [*B. licheniformis*]; ACY72380: cellulose hydrolase [*B. licheniformis*].**

ARW53264.1 -----MSGQKT------------------------------------------------- 6

NVB33365.1 ------------MKQKAFFKMKALCLAFLVIFSMSIASFSEKTRAASAEEYPHNYAELVQ 48

WP_217903639.1 ------MGRKQYVKQKAFFKMKALCLAFLVIFSMSIASFSEKTRAASAEEYPHNYAELVQ 54

ARC67662.1 ------------------------------------------------------------ 0

ARW53955.1 ------------------------------------------------------------ 0

ARC73451.1 ------------------------------------------------------------ 0

WP_025807828.1 ------------MKQKVFLKMKALCLALLVIFSMSIASFSEKTRAASAEEYPHNYAELLQ 48

WP_144619695.1 ------------MKQKVFLKMKALCLALLVIFSMSIASFSEKTRAASAEEYPHNYAELLQ 48

TWJ98598.1 MILTNMMGSKQYVKQKVFLKMKALCLALLVIFSMSIASFSEKTRAASAEEYPHNYAELLQ 60

WP_134778766.1 ------------MKQKVFLKMKALCLALLVIFSMSIASFSEKTRAASAEEYPHNYAELLQ 48

WP_011197979.1 ------------MKQKAFLKMKALCLALLVIFSMSIASFSEKTRAASAEEYPHNYAELLQ 48

WP_223045678.1 --------------------MKALCLALLVIFSMSIASFSEKTRAASAEEYPHNYAELLQ 40

AAR29083.1 ---------------------------------MSIASFSEKTRAASAEEYPHNYAELLQ 27

AHE15442.1 --------------------MKALCLALLVIFSMSIASFSEKTRAASAEEYPHNYAELLQ 40

QGA89116.1 -------------------------LALLVIFSMSIASFSEKTRAASAEEYPHNYAELLQ 35

ACY72380.1 --------------------MKALCLALLVIFSMSIASFSEKTRAASAEEYPHNYAELLQ 40

ARW53264.1 ------------------------------------------------------------ 6

NVB33365.1 KSLLFYEAQRSGRLPENSRLNWRGDSGLEDGKDVGLDLTGGWYDAGDHVKFGLPMAYSVA 108

WP_217903639.1 KSLLFYEAQRSGRLPENSRLNWRGDSGLEDGKDVGLDLTGGWYDAGDHVKFGLPMAYSVA 114

ARC67662.1 ---------------------------MRTEKTVGLDLTGGWYDAGDHVKFGLPMAYSAA 33

ARW53955.1 ---------------------------MRTEKTVGLDLTGGWYDAGDHVKFGLPMAYSAA 33

ARC73451.1 ---------------------------MRTEKTVGLDLTGGWYDAGDHVKFGLPMAYSAA 33

WP_025807828.1 KSLLFYEAQRSGRLPENSRLNWRGDSGLEDGKDVGLDLTGGGYDAGDHVKFGLPMAYSAA 108

WP_144619695.1 KSLLFYEAQRSGRLPENSRLNWRGDSGLEDGKDVGLDLTGGWYDAGDHVKFGLPMAYSAA 108

TWJ98598.1 KSLLFYEAQRSGRLPENSRLNWRGDSGLEDGKDVGLDLTGGWYDAGDHVKFGLPMAYSAA 120

WP_134778766.1 KSLLFYEAQRSGRLPENSRLNWRGDSGLEDGKDVGLDLTGGWYDAGDHVKFGLPMAYSAA 108

WP_011197979.1 KSLLFYEAQRSGRLPENSRLNWRGDSGLEDGKDVGLDLTGGWYDAGDHVKFGLPMAYSAA 108

WP_223045678.1 KSLLFYEAQRSGRLPENSRLNWRGDSGLEDGKDVGLDLTGGWYDAGDHVKFGLPMAYSAA 100

AAR29083.1 KSLLFYEAQRSGRLPENSRLNWRGDSGLEDGKDVGLDLTGGWYDAGDHVKFGLPMAYSAA 87

AHE15442.1 KSLLFYEAQRSGRLPENSRLNWRGDSGLEDGKDVGLDLTGGWYDAGDHVKFGLPMAYSAA 100

QGA89116.1 KSLLFYEAQRSGRLPENSRLNWRGDSGLEDGKDVGLDLTGGWYDAGDHVKFGLPMAYSAA 95

ACY72380.1 KSLLFYEAQRSGRLPENSRLNWRGDSGLEDGKDVGLDLTGGWYDAGDHVKFGLPMAYSAA 100

ARW53264.1 ------------------------------------------------------------ 6

NVB33365.1 ILSWSVYEYRDAYKESGQLDAALGNIKWATDYFLKAHTAPYELWGQVGNGAQDHAWWGPA 168

WP_217903639.1 ILSWSVYEYRDAYKESGQLDAALGNIKWATDYFLKAHTAPYELWGQVGNGAQDHAWWGPA 174

ARC67662.1 ILSWSVYEYRDAYKESGQLDAALDNIKWATDYFLKAHTAPYELWGQVGNGALDHAWWGPA 93

ARW53955.1 ILSWSVYEYRDAYKESGQLDAALDNIKWATDYFLKAHTAPYELWGQVGNGALDHAWWGPA 93

ARC73451.1 ILSWSVYEYRDAYKESGQLDAALDNIKWATDYFLKAHTAPYELWGQVGNGALDHAWWGPA 93

WP_025807828.1 ILSWSVYEYRDAYKESGQLDAALDNIKWATDYFLKAHTAPYELWGQVGNGALDHAWWGPA 168

WP_144619695.1 ILSWSVYEYRDAYKESGQLDAALDNIKWATDYFLKAHTAPYELWGQVGNGALDHAWWGPA 168

TWJ98598.1 ILSWSVYEYRDAYKESGQLDAALDNIKWATDYFLKAHTAPYELWGQVGNGALDHAWWGPA 180

WP_134778766.1 ILSWSVYEYRDAYKESGQLDAALDNIKWATDYFLKAHTAPYELWGQVGNGALDHAWWGPA 168

WP_011197979.1 ILSWSVYEYRDAYKESGQLDAALDNIKWATDYFLKAHTAPYELWGQVGNGALDHAWWGPA 168

WP_223045678.1 ILSWSVYEYRDAYKESGQLDAALDNIKWATDYFLKAHTAPYELWGQVGNGALDHAWWGPA 160

AAR29083.1 ILSWSVYEYRDAYKESGQLDAALDNIKWATDYFLKAHTAPYELWGQVGNGALDHAWWGPA 147

AHE15442.1 ILSWSVYEYRDAYKESGQLDAALDNIKWATDYFLKAHTAPYELWGQVGNGALDHAWWGPA 160

QGA89116.1 ILSWSVYEYPDAYKESGQLDAALDNIKWATDYFLKAHTAPYELWGQVGNGALDHAWWGPA 155

ACY72380.1 ILSWSVYEYPDAYKESGQLDAALDNIKWATDYFLKAHTAPYELWGQVGNGALDHAWWGPA 160

ARW53264.1 --------------------------M--------------------------------- 7

NVB33365.1 EVMPMERPAYKIDAGCPGSDLAGGTAAALASASIIFKPTDSSYSEKLLAHAKQLYDFADR 228

WP_217903639.1 EVMPMERPAYKIDAGCPGSDLAGGTAAALASASIIFKPTDSSYSEKLLAHAKQLYDFADR 234

ARC67662.1 EVMPMKRPAYKIDAGCPGSDLAGGTAAALASASIIFKPTDSSYSEKLLAHAKQLYDFADR 153

ARW53955.1 EVMPMKRPAYKIDAGCPGSDLAGGTAAALASASIIFKPTDSSYSEKLLAHAKQLYDFADR 153

ARC73451.1 EVMPMKRPAYKIDAGCPGSDLAGGTAAALASASIIFKPTDSSYSEKLLAHAKQLYDFADR 153

WP_025807828.1 EVMPMKRPAYKIDAGCPGSDLAGGTAAALASASIIFKPTDSSYSEKLLAHAKQLYDFADR 228

WP_144619695.1 EVMPMKRPAYKIDAGCPGSDLAGGTAAALASASIIFKPTDSSYSEKLLAHAKQLYDFADR 228

TWJ98598.1 EVMPMKRPAYKIDAGCPGSDLAGGTAAALASASIIFKPTDSSYSEKLLAHAKQLYDFADR 240

WP_134778766.1 EVMPMKRPAYKIDAGCPGSDLAGGTAAALASASIIFKPTDSSYSEKLLAHAKQLYDFADR 228

WP_011197979.1 EVMPMKRPAYKIDAGCPGSDLAGGTAAALASASIIFKPTDSSYSEKLLAHAKQLYDFADR 228

WP_223045678.1 EVMPMKRPAYKIDAGCPGSDLAGGTAAALASASIIFKPTDSSYSEKLLAHAKQLYDFADR 220

AAR29083.1 EVMPMKRPAYKIDAGCPGSDLAGGTAAALASASIIFKPTDSSYSEKLLAHAKQLYDFADR 207

AHE15442.1 EVMPMKRPAYKIDAGCPGSDLAGGTAAALASASIIFKPTDSSYSEKLLAHAKQLYDFADR 220

QGA89116.1 EVMPMKRPAYKIDAGCPGSDLAGGTAAALASASIIFKPTDSSYSEKLLAHAKQLYDFADR 215

ACY72380.1 EVMPMKRPAYKIDAGCPGSDLAGGTAAALASASIIFKPTDSSYSEKLLAHAKQLYDFADR 220

ARW53264.1 -----------------------------------------ELIKELVSI---PSPTG-- 21

NVB33365.1 YRGKYSDCITDAQQYYNSWSGYKDELTWGAVWLYLATDEQQYLDKALASVSDWGDPASWP 288

WP_217903639.1 YRGKYSDCITDAQQYYNSWSGYKDELTWGAVWLYLATDEQQYLDKALASVSDWGDPASWP 294

ARC67662.1 YRGKYSDCITDAQQYYNSWSGYKDELTWGAVWLYLATEEQQYLDKALASVSDWGDPANWP 213

ARW53955.1 YRGKYSDCITDAQQYYNSWSGYKDELTWGAVWLYLATEEQQYLDKALASVSDWGDPANWP 213

ARC73451.1 YRGKYSDCITDAQQYYNSWSGYKDELTWGAVWLYLATEEQQYLDKALASVSDWGDPANWP 213

WP_025807828.1 YRGKYSDCITDAQQYYNSWSGYKDELTWGAVWLYLATEEQQYLDKALASVSDWGDPANWP 288

WP_144619695.1 YRGKYSDCITDAQQYYNSWSGYKDELTWGAVWLYLATEEQQYLDKALASVSDWGDPANWP 288

TWJ98598.1 YRGKYSDCITDAQQYYNSWSGYKDELTWGAVWLYLATEEQQYLDKALASVSDWGDPANWP 300

WP_134778766.1 YRGKYSDCITDAQQYYNSWSGYKDELTWGAVWLYLATEEQQYLDKALASVSDWGDPANWP 288

WP_011197979.1 YRGKYSDCITDAQQYYNSWSGYKDELTWGAVWLYLATEEQQYLDKALASVSDWGDPANWP 288

WP_223045678.1 YRGKYSDCITDAQQYYNSWSGYKDELTWGAVWLYLATEEQQYLDKALASVSDWGDPANWP 280

AAR29083.1 YRGKYSDCITDAQQYYNSWSGYKDELTWGAVWLYLATEEQQYLDKALASVSDWGDPANWP 267

AHE15442.1 YRGKYSDCITDAQQYYNSWSGYKDELTWGAVWLYLATEEQQYLDKALASVSDWGDPANWP 280

QGA89116.1 YRGKYSDCITDAQQYYNSWSGYKDELTWGAVWLYLATEEQQYLDKALASVSDWGDPANWP 275

ACY72380.1 YRGKYSDCITDAQQYYNSWSGYKDELTWGAVWLYLATEEQQYLDKALASVSDWGDPANWP 280

ARW53264.1 ----NTYDIIDYINRLLEKEGVETRHNRKGGLIATIPGSDKSRHRMLTAHVDTLGAMV-K 76

NVB33365.1 YRWTLSWDDVTYGAQLLLARL-----TNDSRFTASVER----NLDYWSTGYKNNGSTERI 339

WP_217903639.1 YRWTLSWDDVTYGAQLLLARL-----TNDSRFTASVER----NLDYWSTGYKNNGSTERI 345

ARC67662.1 YRWTLSWDDVTYGAQLLLARL-----TNDSRFVKSVER----NLDYWSTGYSHNGSIERI 264

ARW53955.1 YRWTLSWDDVTYGAQLLLARL-----TNDSRFVKSVER----NLDYWSTGYSHNGSIERI 264

ARC73451.1 YRWTLSWDDVTYGAQLLLARL-----TNDSRFVKSVER----NLDYWSTGYSHNGSIERI 264

WP_025807828.1 YRWTLSWDDVTYGAQLLLARL-----TNDSRFVKSVER----NLDYWSTGYSHNGSIERI 339

WP_144619695.1 YRWTLSWDDVTYGAQLLLARL-----TKDSRFVKSVER----NLDYWSTGYSHNGSIERI 339

TWJ98598.1 YRWTLSWDDVTYGAQLLLARL-----TNDSRFVKSVER----NLDYWSTGYSHNGSIERI 351

WP_134778766.1 YRWTLSWDDVTYGAQLLLARL-----TNDSRFVKSVER----NLDYWSTGYSHNGSIERI 339

WP_011197979.1 YRWTLSWDDVTYGAQLLLARL-----TNDSRFVKSVER----NLDYWSTGYSHNGSIERI 339

WP_223045678.1 YRWTLSWDDVTYGAQLLLARL-----TNDSRFVKSVER----NLDYWSTGYSHNGSIERI 331

AAR29083.1 YRWTLSWDDVTYGAQLLLARL-----TNDSRFVKSVER----NLDYWSTGYSHNGSIERI 318

AHE15442.1 YRWTLSWDDVTYGAQLLLARL-----TNDSRFVKSVER----NLDYWSTGYSHNGSIERI 331

QGA89116.1 YRWTLSWDDVTYGAQLLLARL-----TNDSRFVKSVER----NLDYWSTGYSHNGSIERI 326

ACY72380.1 YRWTLSWDDVTYGAQLLLARL-----TNDSRFVKSVER----NLDYWSTGYSHNGSIERI 331

::* : * :** . .... : :: . :: . *:

ARW53264.1 EIKPNGRLKIDLIGGFNYNSIEGEYCNIETASGKTYTGTILMHQTSVHVYKDAGKAERNQ 136

NVB33365.1 TYTPGGLAWLEQWGALRYASNAA------------FLAFVY-----SDWVDDTEKAKR-Y 381

WP_217903639.1 TYTPGGLAWLEQWGALRYASNAA------------FLAFVY-----SDWVDDTEKAKR-Y 387

ARC67662.1 TYTPGGLALLEQWGSLRYASNAA------------FLAFVY-----SDWVD-TEKAKR-Y 305

ARW53955.1 TYTPGGLALLEQWGSLRYASNAA------------FLAFVY-----SDWVD-TEKAKR-Y 305

ARC73451.1 TYTPGGLALLEQWGSLRYASNAA------------FLAFVY-----SDWVD-TEKAKR-Y 305

WP_025807828.1 TYTPGGLAWLEQWGSLRYASNAA------------FLAFVY-----SDWVD-TEKAKR-Y 380

WP_144619695.1 TYTPGGLAWLEQWGSLRYASNAA------------FLAFVY-----SDWVD-TEKAKR-Y 380

TWJ98598.1 TYTPGGLAWLEQWGSLRYASNAA------------FLAFVY-----SDWVD-TEKAKR-Y 392

WP_134778766.1 TYTPGGLALLEQWGSLRYASNAA------------FLAFVY-----SDWVD-TEKAKR-Y 380

WP_011197979.1 TYTPGGLAWLEQWGSLRYASNAA------------FLAFVY-----SDWVD-TEKAKR-Y 380

WP_223045678.1 TYTPGGLAWLEQWGSLRYASNAA------------FLAFVY-----SDWVD-TEKAKR-Y 372

AAR29083.1 TYTPGGLAWLEQWGSLRYASNAA------------FLAFVY-----SDWVD-TEKAKR-Y 359

AHE15442.1 TYTPGGLAWLEQWGSLRYASNAA------------FLAFVY-----SDWVD-TEKAKR-Y 372

QGA89116.1 TYTPGGLAWLEQWGSLRYASNAA------------FLAFVY-----SDWVD-TEKAKR-Y 367

ACY72380.1 TYTPGGLAWLEQWGSLRYASNAA------------FLAFVY-----SDWVD-TEKAKR-Y 372

.*.* :: *.:.* * . : . : . . : **:*

* * *.*: .*:.

ARW53264.1 KNMEVRIDEPV-NSEEETRALGINVGDFISFDPRVEITPSGFIKSRHLDDKASVALLIEL 195

NVB33365.1 RDFAVRQMQYMLGDNPQQRSFVVGYGKNPPKHPHHRTAHGSWADQMNVPENHRHTLYGAL 441

WP_217903639.1 RDFAVRQMQYMLGDNPQQRSFVVGYGKNPPKHPHHRTAHGSWADQMNVPENHRHTLYGAL 447

ARC67662.1 RDFAVRQTEYMLGDNPQQRSFVVGYGKNPPKHPHHRTAHGSWANQMNVPENHRHTLYGAL 365

ARW53955.1 RDFAVRQTEYMLGDNPQQRSFVVGYGKNPPKHPHHRTAHGSWANQMNVPENHRHTLYGAL 365

ARC73451.1 RDFAVRQTEYMLGDNPQQRSFVVGYGKNPPKHPHHRTAHGSWANQMNVPENHRHTLYGAL 365

WP_025807828.1 RDFAVRQTEYMLGDNPQQRSFVVGYGKNPPKHPHHRTAHGSWANQMNVPENHRHTLYGAL 440

WP_144619695.1 RDFAVRQTEYMLGDNPQQRSFVVGYGKNPPKHPHHRTAHGSWANQMNVPENHRHTLYGAL 440

TWJ98598.1 RDFAVRQTEYMLGDNPQQRSFVVGYCKNPPKHPHHRTAHGSWANQMNVPENHRHTLYGAL 452

WP_134778766.1 RDFAVRQTEYMLGDNPQQRSFVVGYGKNPPKHPHHRTAHGSWANQMNVPENHRHTLYGAL 440

WP_011197979.1 RDFAVRQTEYMLGDNPQQRSFVVGYGKNPPKHPHHRTAHGSWANQMNVPENHRHTLYGAL 440

WP_223045678.1 RDFAVRQTEYMLGDNPQQRSFVVGYGKNPPKHPHHRTAHGSWANQMNVPENHRHTLYGAL 432

AAR29083.1 RDFAVRQTEYMLGDNPQQRSFVVGYGKNPPKHPHHRTAHGSWANQMNVPENHRHTLYGAL 419

AHE15442.1 RDFAVRQTEYMLGDNPQQRSFVVGYGKNPPKHPHHRTAHGSWANQMNVPENHRHTLYGAL 432

QGA89116.1 RDFAVRQTEYMLGDNPQQRSFVVGYGKNPPKHPHHRTAHGSWANQMNVPENHRHTLYGAL 427

ACY72380.1 RDFAVRQTEYMLGDNPQQRSFVVGYGKNPPKHPHHRTAHGSWANQMNVPENHRHTLYGAL 432

::: ** : : ..: : *:: :. . .*: . : ..: .. :: :: :* *

ARW53264.1 -------------IRTINTEGMTLPYTTHFLISNNEEIGYGGNSNIPPETVEYLAVDMGA 242

NVB33365.1 VGGPGRDDSYRDDITDYVSNEVAIDYNAAFTGNVAKMFQLFGKGHVPLPDFP-------- 493

WP_217903639.1 VGGPGRDDSYRDDITDYVSNEVAIDYNAAFTGNVAKMFQLFGKGHVPLPDFP-------- 499

ARC67662.1 VGGPGRDDSYRDDITDYASNEVAIDYNAAFTGNVAKMFQLFGKGHVPLPDFP-------- 417

ARW53955.1 VGGPGRDDSYRDDITDYASNEVAIDYNAAFTGNVAKMFQLFGKGHVPLPDFP-------- 417

ARC73451.1 VGGPGRDDSYRDDITDYASNEVAIDYNAAFTGNVAKMFQLFGKGHVPLPDFP-------- 417

WP_025807828.1 VGGPGRDDSYRDDITDYASNEVAIDYNAAFTGNVAKMFQLFGKGHVPLPDFP-------- 492

WP_144619695.1 VGGPGRDDSYRDDITDYASNEVAIDYNAAFTGNVAKMFQLFGKGHVPLPDFP-------- 492

TWJ98598.1 VGGPGRDDSYRDDITDYASNEVAIDYNAAFTGNVAKMFQLFGKGHVPLPDFP-------- 504

WP_134778766.1 VGGPGRDDSYRDDITDYASNEVAIDYNAAFTGNVAKMFQLFGKGHVPLPDFP-------- 492

WP_011197979.1 VGGPGRDDSYRDDITDYASNEVAIDYNAAFTGNVAKMFQLFGKGHVPLPDFP-------- 492

WP_223045678.1 VGGPGRDDSYRDDITDYASNEVAIDYNAAFTGNVAKMFQLFGKGHVPLPDFP-------- 484

AAR29083.1 VGGPGRDDSYRDDITDYASNEVAIDYNAAFTGNVAKMFQLFGKGHVPLPDFP-------- 471

AHE15442.1 VGGPGRDDSYRDDITDYASNEVAIDYNAAFTGNVAKMFQLFGKGHVPLPDFP-------- 484

QGA89116.1 VGGPGRDDSYRDDITDYASNEVAIDYNAAFTGNVAKMFQLFGKGHVPLPDFP-------- 479

ACY72380.1 VGGPGRDDSYRDDITDYASNEVAIDYNAAFTGNVAKMFQLFGKGHVPLPDFP-------- 484

* :: ::: *.: * . : : *:.::* .

ARW53264.1 IGDGQSTDEYTVSICVKDASGPYHYGLRKRLTALCEAHGIDYKLDIYPYYGSDASAAVKA 302

NVB33365.1 -EKEIPEDEYFAEASINSSGNSY-TEIRAQLNNRSGWPAKKTDQLSFRYYV-DLTEAVEA 550

WP_217903639.1 -EKEIPEDEYFAEASINSSGNSY-TEIRAQLNNRSGWPAKKTDQLSFRYYV-DLTEAVEA 556

ARC67662.1 -EKETPEDEYFAEASINSSGNSY-TEIRAQLNNRSGWPAKKTDQLSFRYYV-DLTEAVEA 474

ARW53955.1 -EKETPEDEYFAEASINSSGNSY-TEIRAQLNNRSGWPAKKTDQLSFRYYV-DLTEAVEA 474

ARC73451.1 -EKETPEDEYFAEASINSSGNSY-TEIRAQLNNRSGWPAKKTDQLSFRYYV-DLTEAVEA 474

WP_025807828.1 -EKETPEDEYFAEASINSSGNSY-TEIRAQLNNRSGWPAKKTDQLSFRYYV-DLTEAVEA 549

WP_144619695.1 -EKETPEDEYFAEASINSSGNSY-TEIRAQLNNRSGWPAKKTDQLSFRYYV-DLTEAVEA 549

TWJ98598.1 -EKETPEDEYFAEASINSSGNSY-TEIRAQLNNRSGWPAKKTDQLSFRYYV-DLTEAVEA 561

WP_134778766.1 -EKETPEDEYFAEASINSSGNSY-TEIRAQLNNRSGWPAKKTDQLSFRYYV-DLTEAVEA 549

WP_011197979.1 -EKETPEDEYFAEASINSSGNSY-TEIRAQLNNRSGWPAKKTDQLSFRYYV-DLTEAVEA 549

WP_223045678.1 -EKETPEDEYFAEASINSSGNSY-TEIRAQLNNRSGWPAKKTDQLSFRYYV-DLTEAVEA 541

AAR29083.1 -EKETPEDEYFAEASINSSGNSY-TEIRAQLNNRSGWPAKKTDQLSFRYYV-DLTEAVEA 528

AHE15442.1 -EKETPEDEYFAEASINSSGNSY-TEIRAQLNNRSGWPAKKTDQLSFRYYV-DLTEAVEA 541

QGA89116.1 -EKETP------------------------------------------------------ 484

ACY72380.1 -EKETPEDEYLADASINSSGNSY-TEIRAQLNNRSGWPAKKTDQLSFRYYV-DLTEAVEA 541

.

ARW53264.1 GHDI----------------------------------VHGLIGPGIDASHAFERTHQS- 327

NVB33365.1 GYSAEDIKVTAGYNEGASVSQLKPYDASKHIYYTEVSFSGVLIYPGGQSAHKKEVQFRLS 610

WP_217903639.1 GYSAEDIKVTAGYNEGASVSQLKPYDASKHIYYTEVSFSGVLIYPGGQSAHKKEVQFRLS 616

ARC67662.1 GYSAEDIKVTAGYNEGASVSELKPHDASKHIYYTEVSFSGVLIYPGGQSAHKKEVQFRLS 534

ARW53955.1 GYSAEDIKVTAGYNEGASVSELKPHDASKHIYYTEVSFSGVLIYPGGQSAHKKEVQFRLS 534

ARC73451.1 GYSAEDIKVTAGYNEGASVSELKPHDASKHIYYTEVSFSGVLIYPGGQSAHKKEVQFRLS 534

WP_025807828.1 GYSAEDIKVTAGYNEGASVSELKPHDASKHIYYTEVSFSGVLIYPGGQSAHKKEVQFRLS 609

WP_144619695.1 GYSAEDIKVTAGYNEGASVSELKPHDASKHIYYTEVSFSGVLIYPGGQSAHKKEVQFRLS 609

TWJ98598.1 GYSAEDIKVTAGYNEGASVSELKPHDASKHIYYTEVSFSGVLIYPGGQSAHKKEVQFRLS 621

WP_134778766.1 GYSAEDIKVTAGYNEGASVSELKPHDASKHIYYTEVSFSGVLIYPGGQSAHKKEVQFRLS 609

WP_011197979.1 GYSAEDIKVTAGYNEGASVSELKPHDASKHIYYTEVSFSGVLIYPGGQSAHKKEVQFRLS 609

WP_223045678.1 GYSAEDIKVTAGYNEGASVSELKPHDASKHIYYTEVSFSGVLIYPGGQSAHKKEVQFRLS 601

AAR29083.1 GYSAEDIKVTAGYNEGASVSELKPHDASKHIYYTEVSFSGVLIYPGGQSAHKKEVQFRLS 588

AHE15442.1 GYSAEDIKVTAGYNEGASVSELKPHDASKHIYYTEVSFSGVLIYPGGQSAHKKEVQFRLS 601

QGA89116.1 ------------------------------------------------------------ 484

ACY72380.1 GYSAEDIKVTAGYNEGASVSELKPHDASKHIYYTEVSFSGVLIYPGGQSAHKNEVQFRLS 601

ARW53264.1 --------------SLEQT--A----KLLYHYVQSDMA------- 345

NVB33365.1 APDGTSFWNPENDHSYQGLSHALLKTRYIPVYDDGRLVFGHEPG- 654

WP_217903639.1 APDGTSFWNPENDHSYQGLSHALLKTRYIPVYDDGRLVFGHEPG- 660

ARC67662.1 APDGTFFWNPENDHSYQGLSHALLKTRYIPVYDDGRLVFGHEPGY 579

ARW53955.1 APDGTSFWNPENDHSYQGLSHALLKTRYIPVYDDGRLVFGHEPGY 579

ARC73451.1 APDGTSFWNPENDHSYQGLSHALLKTRYIPVYDDGRLVFGHEPGY 579

WP_025807828.1 APDGTSFWNPENDHSYQGLSHALLKTRYIPVYDDGRLVFGHEPGY 654

WP_144619695.1 APDGTSFWNPENDHSYQGLSHALLKTRYIPVYDDGRLVFGHEPGY 654

TWJ98598.1 APDGTSFWNPENDHSYQGLSHALLKTRYIPVYDDGRLVFGHEPGY 666

WP_134778766.1 APDGTSFWNPENDHSYQGLSHALLKTRYIPVYDDGRLVFGHEPGY 654

WP_011197979.1 APDGTSFWNPENDHSYQGLSHALLKTRYIPVYDDGRLVFGHEPGY 654

WP_223045678.1 APDGTSFWNPENDHSYQGLSHALLKTRYIPVYDDGRLVFGHEPGY 646

AAR29083.1 APDGTSFWNPENDHSYQGLSHALLKTRYIPVYDDGRLVFGHEPGY 633

AHE15442.1 APDGTSFWNPENDHWYQGLSHALLKTRYIPTAAGQRLVFGHEPGY 646

QGA89116.1 --------------------------------------------- 484

ACY72380.1 APDGTSFWNPENDHSYQGLSHALLKTRYIPVYDDGRLVFGHEPGY 646
